# Inherited Thrombocytopenia: Update on Genes and Genetic Variants Which may be Associated With Bleeding

**DOI:** 10.3389/fcvm.2019.00080

**Published:** 2019-06-19

**Authors:** Ibrahim Almazni, Rachel Stapley, Neil V. Morgan

**Affiliations:** Institute of Cardiovascular Sciences, College of Medical and Dental Sciences, University of Birmingham, Birmingham, United Kingdom

**Keywords:** inherited thrombocytopenia, platelets, megakaryocytes, genes, mutations, bleeding

## Abstract

Inherited thrombocytopenia (IT) is comprised of a group of hereditary disorders characterized by a reduced platelet count as the main feature, and often with abnormal platelet function, which can subsequently lead to impaired haemostasis. Inherited thrombocytopenia results from genetic mutations in genes implicated in megakaryocyte differentiation and/or platelet formation and clearance. The identification of the underlying causative gene of IT is challenging given the high degree of heterogeneity, but important due to the presence of various clinical presentations and prognosis, where some defects can lead to hematological malignancies. Traditional platelet function tests, clinical manifestations, and hematological parameters allow for an initial diagnosis. However, employing Next-Generation Sequencing (NGS), such as Whole Genome and Whole Exome Sequencing (WES) can be an efficient method for discovering causal genetic variants in both known and novel genes not previously implicated in IT. To date, 40 genes and their mutations have been implicated to cause many different forms of inherited thrombocytopenia. Nevertheless, despite this advancement in the diagnosis of IT, the molecular mechanism underlying IT in some patients remains unexplained. In this review, we will discuss the genetics of thrombocytopenia summarizing the recent advancement in investigation and diagnosis of IT using phenotypic approaches, high-throughput sequencing, targeted gene panels, and bioinformatics tools.

## Introduction

**Figure 1:**
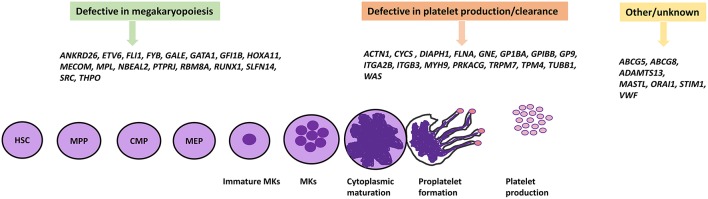
IT causative genes involved in megakaryopoiesis, platelet formation, and others. The differentiation of platelets from HSCs proceeds by multiple different cell lineages which involve many genes encoding a number of transcription factors and proteins. Genetic defects in these genes have been shown to cause IT. HSC, Hematopoietic stem cell; MPP, Multi-Potent Progenitor; CMP, Common myeloid progenitor; MEP, Megakaryocyte-erythroid progenitor.

Platelets are small anucleate cells produced by megakaryocytes in the bone marrow (BM) where they circulate in the blood to protect the integrity of blood vessels. They play an important role in normal haemostasis to prevent excessive bleeding at the site of blood vessel injury (1, 2). Inherited Thrombocytopenias (ITs) are a heterogeneous group of disorders characterized by low platelet counts, often manifesting as bleeding diathesis which subsequently result in impaired haemostasis (3). In 1948, the disease inheritance pattern of one IT was initially discovered in a disorder called Bernard-Soulier syndrome (BSS) (4). Since then, the advancement in clinical and scientific research has led to an increased understanding of the molecular defects in patients with ITs. These defects are variable in severity, ranging from severe bleeding, which can be recognized within a few weeks after birth, to mild bleeding that may remain undiagnosed until incidental recognition during routine blood testing in adulthood (5). They manifest with different symptoms including epistaxis, easy bruising, petechiae, prolonged bleeding from cuts, gum bleeding, excessive bleeding after surgery, hematuria, and menorrhagia in women (6, 7). As bleeding is considered the main clinical complication for patients with IT, some patients with common ITs have the propensity to develop other disorders such as hematological malignancies and kidney failure (4, 8). Although there are other causes of thrombocytopenia, such as infections and immune disorders, IT is primarily caused by mutations in genes involved in megakaryocyte differentiation, maturation and platelet release (9). Since the last decade, next generation sequencing technologies, namely Whole Exome Sequencing (WES) and Whole Genome Sequencing (WGS) coupled with conventional Sanger sequencing and *in-silico* bioinformatic tools have been used in parallel to uncover novel genes with a pivotal role in megakaryocyte biology and platelet biogenesis (10, 11). To date, 40 genes have been reported to cause different forms of IT, which reflects the immense difficulty in identifying a single causative gene, particularly when accompanied by other hematological disorders [Table 1; Figure 1; (27, 28, 54)]. These genetic forms have various clinical manifestations, phenotypic presentations and sometimes associated with secondary qualitative defects in platelet function (7). Diverse platelet phenotypes mean there are several approaches in which they can be characterized. One such way is to classify genes based on their influence on megakaryocyte differentiation, platelet production, and removal (54), and will be discussed below. However, despite these advancements, nearly 50% of patients with IT of unknown genetic etiology still remain undiagnosed (6, 10).

**Table 1:** The direct genetic causes of inherited thrombocytopenia and their associated syndromes.

**Area of mutational effect**	**Gene**	**Syndrome**	**Other syndromic and features**	**References**
Megakaryopoiesis	*ANKRD26*	*ANKRD26*-related thrombocytopenia	Predisposition to leukemia. Reduction of platelet α-granules. Normal *in vitro* platelet aggregation and mean platelet volume. Some patients have high level of hemoglobin and leukocyte.	(12, 13)
	ETV6	*ETV6*-related thrombocytopenia	Leukemia predisposition. High erythrocyte mean corpuscular volume (MCV). Some patients have elevated red cell MCV.	(14)
	*FLI1*	Paris-Trousseau thrombocytopenia/Jacobsen syndrome	Abnormal development of heart and face. Intellectual disabilities. Large α-granules. Abnormal MKs morphology. Normal RBCs and WBCs counts. Moderate thrombocytopenia.	(15)
	*FYB*	*FYB*-related thrombocytopenia	Small platelets. Reduction of mature MKs in BM. Significant bleeding tendency. Normal WBCs count. Low mean platelet volume MPV. Mild iron deficiency anemia.	(16)
	*GATA1*	GATA1-related disease: X-linked thrombocytopenia (XLT) and X-linked thrombocytopenia with thalassemia (XLTT)	Dyserythropoietic anemia. Macrothrombocytopenia. Beta-thalassemia Congenital erythropoietic porphyria. Erythrocyte abnormalities. Splenomegaly.	(17)
	*GFI1B*	Macrothrombocytopenia and platelet function defects	Macrothrombocytopenia. Red cell anisopoikilocytosis Platelet dysfunction. Reduction of platelet α-granules.	(18)
	*HOXA11*	Amegakaryocytic thrombocytopenia with radio-ulnar synostosis	Bilateral radioulnar synostosis. Severe bone marrow failure. Cardiac and renal malformations. B-cell deficiency. Hearing loss. Clinodactyly. Some patients show skeletal anomalies. Some patients have developed pancytopenia.	(19)
	*MECOM*	Congenital amegakaryocytic thrombocytopenia and radioulnar synostosis		(20)
	*MPL*	Congenital amegakaryocytic thrombocytopenia (CAMT)	Absence or reduced of MKs in BM. No physical anomalies. Development to BM aplasia in infancy.	(21)
	*NBEAL2*	Gray platelet syndrome	Impaired platelet function. Severe reduction of platelet α-granules contents. Large platelets. Development of myelofibrosis and splenomegaly in some patients. Abnormalities in megakaryocyte development.	(22)
	*RBM8A*	Thrombocytopenia-absent radius syndrome	Bilateral radial aplasia. Elevated hemoglobin level in patients with 5′UTR SNP. Normal WBCs count and some patients have leucocytosis and eosinophilia. Anemia. Skeletal, urogenital, kidney, and heart defects. Reduced MKs in BM.	(23)
	*RUNX1*	Familial platelet disorder with propensity to acute myelogenous leukemia (FPD/AML)	Platelet defects. Variable platelet counts. Reduction in dense granule secretion observed in secondary qualitative abnormality. Myelodysplasia. Reduced response to several platelet agonists.	(8)
	*SLFN14*	*SLFN14*-related thrombocytopenia	Giant platelets. Decreased ATP secretion. Reduced number of dense granules.	(24)
	*SRC*	SRC-related thrombocytopenia	Myelofibrosis, bleeding, and bone pathologies. Hypercellular bone marrow with trilineage dysplasia. Platelets are dysmorphic and variable in size. Paucity of α-granules. Splenomegaly, congenital facial dysmorphism. Abundant vacuoles.	(25)
	*THPO*	Inherited thrombocytopenia from monoallelic *THPO* mutation	Bone marrow aplasia. Normal or enlarged platelet morphology.	(26)
	*PTPRJ*	Inherited thrombocytopenia	Syndromic thrombocytopenia characterized by spontaneous bleeding, small-sized platelets. Impaired platelet function.	(27)
	*GALE*	Inherited thrombocytopenia	Dysplastic megakaryocytes. Some patients have mild anemia and febrile neutropenia. Big and pale platelets. Galactosemia, hypotonia, seizures, jaundice, galactosuria, and hepatomegaly.	(28)
Platelet production/clearance	*ACTIN1*	*ACTN1*-related thrombocytopenia	Congenital macrothrombocytopaenia. Anisocytosis. Absent or mild bleeding diathesis.	(29)
	*CYCS*	CYCS-related thrombocytopenia	Normal platelet size and volume.	(30)
	*GNE*	*GNE* myopathy with congenital thrombocytopenia	Rimmed vacuoles. Hematological complications are rare. Proteinuria and hematuria in some patients. Membranoproliferative glomerulonephritis. Platelets size are normal to large.	(31–33)
	*GP1BA*	Bernard-Soulier Syndrome (BSS) + platelet type von-Willebrand disease (PTvWD)	Macrothrombocytopaenia. Severe bleeding tendency with platelet function defect. Platelet anisocytosis.	(34–36)
	*GPIBB*			
	*GP9*			
	*ITGA2B*	Glanzmann thrombasthenia	Impaired platelet function.	(37, 38)
	*ITGB3*			
	*MYH9*	*MYH9*-related disease (MYH9-RD)	Congenital macrothrombocytopaenia. Mild bleeding tendency. Development of kidney dysfunction, deafness, cataracts, and Döhle-like bodies. Elevated liver enzymes.	(39)
	*PRKACG*	*PRKACG*-related thrombocytopenia	Giant platelet. Impaired platelet function.	(40)
	*TRPM7*	TRPM7-related thrombocytopenia	Macrothrombocytopaenia. Atrial fibrillation.	(41)
	*TPM4*	Tropomyosin 4-related thrombocytopenia	Macrothrombocytopaenia. All other blood cell counts are normal. Mild effect on platelet function.	(42)
	*TUBB1*	*TUBB1*-related thrombocytopenia	Congenital macrothrombocytopaenia.	(43)
	*WAS*	Wiskott-Aldrich syndrome, X-linked thrombocytopenia (XLT)	Mild or severe immunodeficiency, hematopoietic malignancies, and eczema. Thrombocytopenia with small platelets. Autoimmune haemolytic anemia.	(44)
	*FLNA*	Filaminopathies A	X-linked dominant form of periventricular nodular heterotopia (FLNA-PVNH) and the otopalatodigital syndrome spectrum of disorders. Hemorrhage and coagulopathy. Abnormal platelet morphology.	(45)
	*DIAPH1*	Macrothrombocytopenia (MTP) and hearing loss	_	(46)
Other/unknown	*ABCG5 ABCG8*	Macrothrombocytopenia associated with sitosterolemia	Xanthomas and pre-mature coronary atherosclerosis due to hypercholesterolemia. Hematologic abnormalities.	(47)
	*ADAMTS13*	Thrombotic thrombocytopenic purpura	Upshaw_Schulman syndrome. Anemia.	(48)
	*STIM1*	Stormorken syndrome and york platelet syndrome	Tubular myopathy and congenital miosis. Severe immune dysfunction.	(49, 50)
	*vWF*	Von Willebrand disease type IIB		(51)
	*ORAI1*	Stormorken syndrome	CRAC channelopathy. Severe combined immunodeficiency, autoimmunity, muscular hypotonia, and ectodermal dysplasia.	(52)
	*MASTL*	Autosomal dominant thrombocytopenia	_	(53)

## IT Genes Associated with Megakaryocyte Differentiation and Maturation

The process of megakaryopoiesis and thrombopoiesis involves a complicated biological series of events. Megakaryocytes, like all blood cells, are derived from the hematopoietic stem cells (HSC) in the bone marrow during the lineage commitment stages. The hematopoietic stem cell differentiation process involves committed precursors that include the common myeloid progenitor (CMP) and the megakaryocyte-erythroid progenitor (MEP). The erythrocyte cells and megakaryocyte cells result from the MEP. Megakaryocyte precursors encompass a maturation process that results in mature polyploid megakaryocytes, and then leads to the formation of pro-platelets (54, 55). The process of megakaryopoiesis and thrombopoiesis involves multiple genes and transcriptions factors (detailed below) which play important roles in megakaryocyte differentiation, platelet formation, and release. IT can result from defects in these genes which present with variable phenotypic display and clinical presentation. As a result of the numerous clinical demonstrations of ITs, they can be characterized based on genes and their role during megakaryocyte differentiation, platelet production, and release (9, 56).

Some ITs result from defective changes from haemopoietic progenitor cells to MKs, leading to reduction or absence in the number of bone marrow MKs. Thrombopoietin (TPO), an acidic glycoprotein, is the main regulator of the megakarypoiesis and thrombopoiesis mechanism in humans, acting through its receptor c-Mpl. It is required for megakaryocytes to fulfill their developmental proliferation and for the subsequent maturation of platelets (57). Affected individuals from a large Micronesian family displayed idiopathic anemia and mild thrombocytopenia as a result of mutations in *TPO* and *MPL* genes (21, 58). The main defective mechanism in several forms of IT is a change in MK maturation which therefore leads to the production of immature and dysfunctional MKs. However, the differentiation and maturation of MKs is regulated by several transcription factors such as *GATA1*. *GATA1* is highly expressed in the erythroid and megakaryocytic lineage, and plays a vital role in the maturation and development of erythroid cells and megakaryocytes (59). X-linked thrombocytopenia with thalassemia and X-linked thrombocytopenia with dyserythropoietic anemia are both caused by mutations in *GATA1*, resulting in impaired MK and erythroid cell maturation. As a consequence, *GATA1*-mutated patients are characterized with large platelets and reduced α-granule contents. They also display a variable degree of anemia and abnormal morphology of red blood cells (60). Additional transcription factors known to be involved in the maturation of megakaryocytes are *RUNX1, ETV6, ANKRD26, FLI-1***, **and the transcriptional repressor *GFI1B* acting by binding to promoter regions in MK expressed genes. Thus, multiple mechanisms in MK and platelet maturation are affected as result of alterations in these genes (61, 62). A previous study identified a point mutation in the third helix of *HOXA11* homeodomain causing an inherited syndrome of congenital amegakaryocytic thrombocytopenia and radio-ulnar synostosis (19). Thrombocytopenia absent radii (TAR) syndrome results from a combination of a microdeletion on Chromosome 1 including the *RBM8A* gene alongside a low frequency non-coding single nucleotide polymorphism (SNP) within the regulatory region of *RBM8A* (23). As a consequence, hematopoietic progenitors from patients with TAR syndrome fail to differentiate into MKs *in vitro* (63). Gray Platelet syndrome is characterized by a deficiency in α granule content which also results in a platelet function defect. It is associated with enlarged platelets and mild thrombocytopenia with moderate to severe bleeding as a result of biallelic mutations in *NBEAL2*, the gene encoding the neurobeachin-like-2 protein (22). Variants in the 5'UTR of *ANKRD26* cause familial thrombocytopenia type-2 (THC2) with propensity to leukemia, which result in loss of RUNX1 and FLI1 binding and prevents gene silencing (12). Moreover, heterozygous variants specifically located in the promoter region between c.-134G and c.-113 region highly affect gene expression. Patients with THC2 are characterized by small MKs with hypolobulated nuclei as a result of dysmegakaryopoiesis (64). A mutation in the *FYB1* gene has recently been identified to cause IT and although the exact mechanism of the mutation is still ambiguous, it has been suggested that thrombocytopenia arises from a reduction of mature MKs in the bone marrow and synthesis of small platelets (16).

## Defects in Proplatelet Formation and Platelet Release

After megakaryopoesis, proplatelets form extensions which lead to **“**budding**”** at the tips and platelet release into the circulation. Mature MKs undergo essential processing by extending long branches called proplatelets via the bone marrow sinusoids, and subsequently release platelets into the blood circulation. These processes are underpinned by cytoskeletal changes and cellular signaling where most causative mutations of IT disrupt the pathway reducing the circulating platelet count (65, 66). Mature polyploid MKs cytoplasm extend long beaded cytoplasmic protrusions, as a result of microtubule sliding. The dimerisation of β1-tubulin with α-tubulin polymerizes into long microtubule bundles inside the MK cortex. A mixed polarity of microtubule bundles runs throughout the extension of proplatelets which are thought to provide fundamental force for microtubule sliding and proplatelet elongation (65). *TUBB1* encodes for β1-tubulin and mutations within *TUBB1* are associated with an autosomal dominant form of IT known as a congenital macrothrombocytopenia (43). WASp is a multidomain protein belonging to a family of actin nucleation-promoting factors (NPFs) which are specifically expressed in hematopoietic cells. WASp plays an important role in actin polymerization by transmission of surface signals via the actin-related protein (Arp)2/3 complex (44, 67). Mutations have been identified in the *WAS* gene which cause a rare X-linked disorder called Wiskott-Aldrich syndrome (WAS). Patients are characterized by micro-thrombocytopenia and immunodeficiency with predispostion to malignancies (68). The transmission of extracellular signals into the cytoskeleton is mediated via membrane bound receptors which have been associated with mutations in IT. One of the main membrane receptors in platelets/MKs is the GP1b-IX-V complex, which binds specifically to Von-Willebrand factor (VWF). This receptor is comprised of four subunits including GP1bα, GP1bβ, GPIX, and GPV. Binding of VWF with GP1bα leads to activation and signal transmission to form the extending proplatelet. Mutations in the encoding genes *GP1BA, GP1BB***, **and *GP9* cause monoallelic and biallelic forms of BSS (69, 70). Other receptors include the receptor for fibrinogen, integrin GPIIb-IIIa, which is encoded by the genes *ITGA2B* and *ITGB3*. Affected mutations in *ITGA2B* and *ITGB3* have been identified to cause Glanzmann thrombasthenia (GT) (71), but patients have a normal platelet count.

## Initial Diagnosis of Hereditary Thrombocytopenia

Identification of the genetic cause in patients with IT is challenging and many patients may be misdiagnosed with acquired thrombocytopenia such as immune thrombocytopenic purpura (ITP). IT can be recognized in patients when a low platelet count has been identified after birth, the presence of familial medical history with similar clinical presentations, evaluation of peripheral blood films, and physical examination. Moreover, the presence of a severe bleeding tendency (in combination with a low platelet count), a lifelong history of diathesis, and evidence of other clinical complications that are typically associated with thrombocytopenia in syndromic forms, and all help to diagnose IT (4, 72). Platelets are involved in other biological roles beyond hemostasis, such as immunity and inflammation (73–75) therefore, mutations in platelet specific genes may cause functional disruption in hemostasis, other biological pathways or both. Furthermore, some proteins are expressed in megakaryocytes and platelets and can be found in other cell types. *GATA1* is a prominent example which involves megakaryopoiesis and erythropoiesis (76). *MYH9* also has an important role in the platelet cytoskeleton and has been found expressed in kidney and inner ear cilia (77). Based on this, inherited bleeding disorders can be classified into three categories including disorders that (i) affect only platelets, (ii) disorders that are associated with syndromic or non-syndromic phenotypes, and (iii) disorders with increased risk of haematologic malignancies. This classification can be used for both diagnostic and prognostic purposes (4, 11).

## Syndromic Disorders Associated With IT

**Figure 2:**
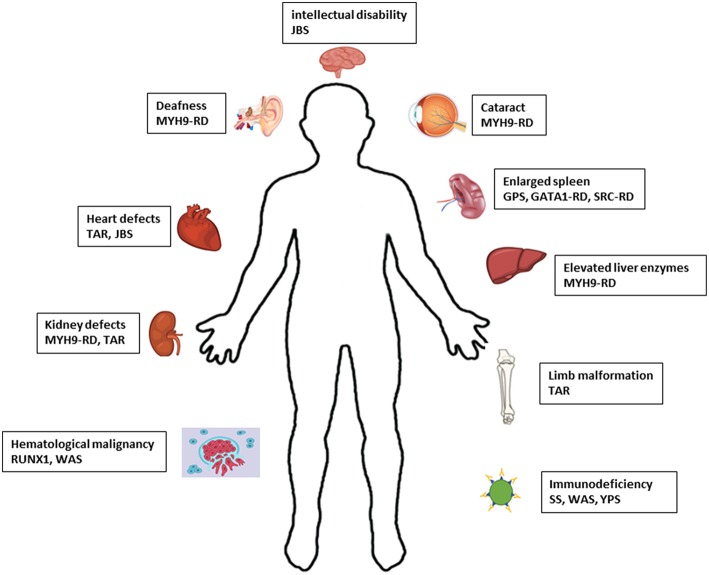
Schematic representation of the defects associated with syndromic IT. JBS, Jacobsen syndrome; RD, related disease; TAR, Thrombocytopenia-absent radius; GPS, Gray platelet syndrome; SS, Stormorken syndrome; WAS, Wiskott-Aldrich syndrome; YPS, York platelet syndrome.

The number of IT forms identified has increased over the last few years since the implementation of NGS. Consequently, it has been shown that the bleeding is not the only clinical phenotype with IT, but patients with some IT forms have propensity to develop more syndromic disorders as result of molecular defects in genes responsible for thrombocytopenia. For instance, hematological malignancies, bone marrow aplasia, skeletal malformation, liver and kidney malfunction, and deafness (Figure 2). The development of these diseases can be more severe for patients than the bleeding itself (4) however, it is still important to recognize if these manifestations are present in the relatives. Some syndromic phenotypes associated with ITs are variable between family members or can arise later in life. For example, development of deafness, kidney malfunction and/or cataract in patients with *MYH9*-RD occur only in adult individuals and it has been reported that patients of the same MYH9-RD pedigrees have variable clinical manifestations (78).

## The Genetic Diagnosis of IT

Some patients with a low platelet count may be falsely diagnosed and receive unnecessary treatments such as immunosuppression and splenectomies and therefore it is paramount that strong evidence must prove that the condition is truly genetic. Genetic diagnosis is a vital approach in providing patients with clinical benefits and prevent unnecessary treatments. Patients with genetic mutations in *RUNX1* have a predisposition to develop hematological malignancies where the genetic information can be used to monitor the patients' hematological parameters very closely. This emphasizes the importance and need for definitive genetic diagnostic tools to provide quick and cost-effective diagnosis for screening patients with IT (6, 8). The molecular basis of ITs has been elucidated since the adoption of Sanger sequencing and linkage analysis in the 1990s. Recently, Sanger sequencing is considered a low throughput and time-consuming approach which can be used initially as a standard tool to investigate patients based on precise clinical findings and phenotype (4). A targeted thrombocytopenia gene specific panel is a useful approach which can be used as initial screening prior to WES. This targeted panel encompasses all known genes associated with IT and their related genes. The aim of using an IT gene specific panel is to filter out patients based on variants in known IT-causal genes and subsequently allowing for WES for patients with unknown genetic etiology (4, 6). The ThromboGenomics project provided a multi-gene high-throughput sequencing platform (HTS) for the diagnosis of heritable bleeding disorders (79). The HTS platform covers approximately 96 genes associated with inherited bleeding, thrombotic, coagulation, and platelet disorders. The panel showed high sensitivity in detecting causative variants in patients who had not been previously investigated at the molecular level. It has a high sensitivity to detect variants in the exonic region as well as many of exonic-intronic boundaries and untranslated regions (UTRs) (6, 79).

## Next Generation Sequencing

Targeted NGS platforms can be efficiently applied to determine the causative genes of IT. As the molecular basis of ITs remain unknown in many patients, WGS or WES may be required which improves the knowledge of ITs at the molecular level. Several national and international consortia have adopted these approaches to identify disease-causing genes associated with IT. The genes *SLFN14, FYB, STIM1, GFI1b*, and *ETV6* are some examples of causative genes detected by these approaches. The results obtained by HTS improves the understanding of the functional role in some causative genes, whose function in platelet production was previously unknown. These techniques will bring substantial benefits to improve our understanding of the molecular mechanisms in megakaryocyte and platelet biogenesis (14, 16, 24, 49, 80, 81). However, distinguishing pathogenic variants from non-pathogenic variants often requires complex functional and cell line studies to prove causality (7).

## Bioinformatic Tools

Bioinformatic tools can be conducted to determine candidate variants from WES or WGS data. A wide range of variants, ~25,000–40,000 variants can be identified per single patient in WES. These variants can be filtered for novelty by direct comparison using a database from the 100,000 Genomes Project, Exon Variant Server (EVS), dbSNP versions, Exome Aggregation Consortium (ExAC) (gnomAD), and in-house databases of whole exomes and/or whole genomes. A database of known platelet-related genes and genes involved in platelet formation, function, lifespan, or death can be compared with the patient's genes in order to narrow the candidate genetic variants down. Variants with MAF (minor allele frequency) ≥0.01 are generally excluded given the rarity of most of these genetic defects in IT. Variants not known to change the amino acid or those that do not have a potential effect on protein, such as synonymous variants and intron variants can also be excluded. Splice site variants occurring >5 base pair away from the exome can be also excluded, although this can potentially result in splicing or regulatory mutations being missed. Comparisons with other affected and unaffected family members on the database can be used to select candidate variants. Also, pathogenicity prediction can be assessed by using different tools such PolyPhen2, Provean, SIFT, Mutation Taster, mRNA expression levels which predict the potential effect of amino acid changes on protein structure and function and also measure the conservation of amino acids among different species. Sapientia is a recently developed clinical diagnostic platform established by Congenica to help clinicians, clinical scientists, and researchers with genetic diagnosis and identification of disease-causing genes, by interrogating the human genome with multiple bioinformatic tools. It can help streamline the process of diagnosis, ensuring patients are receiving accurate information and treatments for their individual platelet or megakaryocyte defect.

## Conclusions

The advances in NGS techniques improves our knowledge about the molecular mechanisms of IT. The major risk factor for patients with ITs is the development of additional syndromic disorders rather than bleeding itself. Due to the polygenic nature of ITs and disorders involved in hematopoiesis, identifying a singular causative gene for platelet and megakaryocyte function is particularly difficult. A combination of whole blood counting and platelet functional assays will highlight the platelet phenotype, however only familial studies and genetic sequencing will help to identify any genetic defect. With the introduction of HTS and various genome browser software such as Sapienta, the diagnosis process has been modernized to highlight candidate variants in known platelet affected genes and reveal variants in novel genes in which hemostatic input remains to be explored. Approaches such as these may be implemented within clinical settings in the future, however, bioinformatic pipelines are yet to be standardized across all facilities. Aside from bioinformatic training and the initial financial burden of installation of the software, there are clear reasons that updating current genetic analysis in hematological disorders benefits healthcare in the wider community. This will ensure IT families obtain a clear diagnosis and receive correct treatment based on their genetically influenced megakaryocyte or platelet defect.

## Author Contributions

IA wrote the manuscript. IA, RS, and NM critically reviewed and edited the review.

### Conflict of Interest Statement

The authors declare that the research was conducted in the absence of any commercial or financial relationships that could be construed as a potential conflict of interest.
